# Long non‐coding RNA SNAI3‐AS1 promotes the proliferation and metastasis of hepatocellular carcinoma by regulating the UPF1/Smad7 signalling pathway

**DOI:** 10.1111/jcmm.14513

**Published:** 2019-07-02

**Authors:** Yarui Li, Dan Guo, Mudan Ren, Yan Zhao, Xin Wang, Yifei Chen, Yaping Liu, Guifang Lu, Shuixiang He

**Affiliations:** ^1^ Department of Gastroenterology The First Affiliated Hospital of Xi'an Jiaotong University Xi'an China

**Keywords:** EMT, hepatocellular carcinoma, metastasis, proliferation, SNAI3‐AS1, UPF1

## Abstract

Emerging evidence has indicated that deregulation of long non‐coding RNAs (lncRNAs) can contribute to the progression of human cancers, including hepatocellular carcinoma (HCC). However, the role and exact mechanism of most lncRNAs in tumours remains largely unknown. In the current study, we found a novel long non‐coding RNA termed SNAI3‐AS1 which was generally up‐regulated in HCC tissues compared with normal control. Higher expression of SNAI3‐AS1 was significantly correlated with shorter overall survival of HCC patients. Knockdown of SNAI3‐AS1 inhibited the proliferation and metastasis of HCC cells in vitro, whereas overexpression of SNAI3‐AS1 promoted the proliferation and metastasis of HCC cells. Further investigations showed that SNAI3‐AS1 could affect HCC tumorigenesis by binding up‐frameshift protein 1 (UPF1), regulating Smad7 expression and activating TGF‐β/Smad pathway. Functionally, SNAI3‐AS1 promoted HCC growth and metastasis by inducing tumour epithelial to mesenchymal transition (EMT). Taken together, these findings showed that SNAI3‐AS1 promotes the progression of HCC by regulating the UPF1 and activating TGF‐β/Smad pathway.

## 1 INTRODUCTION

Hepatocellular carcinoma (HCC) is the dominant histological type of primary malignancies in liver and the second highest leading cause of cancer‐related death worldwide.^1^ Although great progress has been made in the diagnosis and treatment of HCC, it still has a high post‐operative recurrence rate due to tumour metastasis and chemoresistance.^2^, ^3^ Therefore, it is urgent to explore potential biomarkers and molecular mechanisms of HCC tumorigenesis, which contribute to early diagnosis and effective therapy.

In recent years, the role of protein‐coding genes in the development and progression of HCC has been extensively studied, and some molecular markers that can be used to determine the prognosis of HCC have been explored.^4^, ^5^, ^6^ However, the molecular mechanism of HCC has not yet been fully elucidated and needs to be further explored. Long non‐coding RNAs (lncRNAs) are a class of RNA transcripts which are more than 200 nucleotides in length and have no protein‐coding potential. New evidence showed that lncRNAs play important regulatory roles in tumorigenesis or cancer progression, hence, lncRNAs have gained more and more attention and may present new opportunities for disease diagnosis and treatment.^7^, ^8^, ^9^ Meanwhile, the number of lncRNAs has been found to be aberrantly expressed in multiple human cancers, including HCC.^10^, ^11^, ^12^, ^13^, ^14^ These aberrant expressed lncRNAs regulate a wide range of important pathophysiological processes which associated with tumorigenesis, metastasis, prognosis. As a new type of regulatory RNA molecule, lncRNA has a diverse subcellular location and plays important roles in many aspects of cell activity. LncRNAs interfere gene expression in transcriptional or post‐transcriptional process by direct or indirect ways.^15^, ^16^ Therefore, studying the role of lncRNAs in HCC may help to further understand HCC carcinogenesis.

In this study, we identified a novel HCC related lncRNA, termed SNAI3 antisense RNA 1 (SNAI3‐AS1) by two human HCC microarray results (GSE58043 and GSE55191), and subsequently examined the role of SNAI3‐AS1 in HCC and the potential mechanisms involved by a retrospective analysis of 46 HCC patients, and by carrying out in vitro experiments to clarify the contribution of SNAI3‐AS1 to the proliferation and metastasis of HCC and its effect on EMT.

## 2 MATERIALS AND METHODS

### 2.1 Clinical samples and cell lines

A total of 46 pairs fresh HCC tissue specimens and matched adjacent non‐malignant tissues (3‐5 cm distal to the edge of tumour) were collected from patients underwent resection of primary HCC at the First Affiliated Hospital of Xi'an Jiaotong University. None of patients received any chemotherapy or radiotherapy treatments before surgery. Written informed consent from all patients and approval of the Hospital Ethic Committees was obtained. Kaplan‐Meier and log‐rank analyses were used for survival analysis. All human HCC cells involved and immortalized human hepatic cell LO2 were purchased from the Type Culture Collection of the Chinese Academy of Sciences. All cells were cultured in DMEM/high glucose (Hyclone), supplemented with 10% foetal bovine serum (FBS, Gibco) and 100 μg/mL streptomycin and 100 U/mL penicillin (Hyclone), and maintained at 37°C in humidified incubator with 5% CO_2_.

### 2.2 RNA isolation, and quantitative real‐time PCR

The total RNA was extracted from tissues or cultured cells according to the instruction of Trizol Reagent (Invitrogen). Then, the cDNAs were synthesized following the protocol of PrimeScript™ RT Master Mix (Takara). Quantitative real‐time PCR was conducted using SYBR Premix Ex TaqTM II (Takara) on Thermal Cycler CFX6 System (BioRad). β‐actin was used as internal reference. The relative expression of genes was calculated using the 2^−ΔΔCt^ method. The primers used in this study were presented in Supplementary Table 1.

### 2.3 Transfection of cell lines

A shRNA targeting SNAI3‐AS1 was designed by Genechem to construct stable SNAI3‐AS1 knockdown cell lines. SNAI3‐AS1 and UPF1 were cloned into the expression vector pCMV (Invitrogen) for overexpression. The small interfering RNA (siRNA) against UPF1 and negative control (NC) were designed by Genepharma. The siRNA sequences were presented in Supplementary Table 1. Transfection assay was conducted according to the instruction of Lipofectamine 2000 (Invitrogen) when the cells reached approximately 60%‐80% confluence.

### 2.4 Cell viability assay (MTT assay)

Cell viability was assessed by MTT (3‐(4,5‐dimethylthiazol‐2‐yl)‐2, 5‐diphenyltetrazolium bromide) assay. Firstly, cells were seeded into 96‐well plates at a density of 5000 cells per well. 24 hours after seeding, transfection was conducted as the reagent's protocol. After transfection for 24 hours, 48 hours, 72 hours, 96 hours, 10 μL of 5 mg/mL MTT was added into each well and then cultured for 4 hours in incubator. The supernatant was then discarded and 150 μL of DMSO was added to dissolve the crystal. The optical density (OD) was measured by EnSpire Multimode Plate Reader (PerkinElmer) at 490 nm. Triplicate experiments were performed for each assay.

### 2.5 Colony formation assay

Transfect the cells with the indicated reagents. After 24 hours of routine incubation, cells were re‐plated at a density of 500 cells/6 cm plates and maintained in DMEM. After 2 weeks, when clones formed by single cell possessed at least 50 cells, the clones were fixed with methanol and stained with 0.1% crystal violet in PBS for 15 minutes. The colony formation was determined by counting the number of stained colonies.

### 2.6 Cell migration and invasion assays

#### 2.6.1 Transwell assay

After 48 hours of transfection, cells were respectively seeded into 8 μm pore size transwell 24‐well chambers (Merck Millipore) coated with Matrigel (BD Biosciences) for invasion assay and non‐coated chambers for migration. A total of 5 × 10^4^ cells/200 μl cells in serum‐free medium were placed into the upper chamber, DMEM medium containing 10% FBS was added to the lower chamber. After incubation for 24 hours, we wiped off the non‐invaded or non‐migrated cells gently on the on the upper membrane. Cells that had invaded or migrated through the membrane were fixed with 95% ethyl alcohol for 10 minutes, followed by crystal violet staining for 15 minutes and washed in PBS. Stained cells were observed under optical microscope and counted.

#### 2.6.2 Wound healing assay

Appropriate cell density is required which need to attain 90% after 24 hours of transfection in 6‐well plates. Wound was scratched by 10 μL sterile tip and then washed off the floated cells. The wound size was measured and photographed at 0 hour, 24 hours and 48 hours.

### 2.7 Western blot analysis

The transfected cells were lysed by using RIPA (Beyotime) supplemented with proteinase and phosphatase inhibitors. Protein samples were loaded for electrophoresis (5% gel for concentration and 10% for separation), and then transferred onto 0.45 μm or 0.22 μm pore size PVDF membrane (Merck Millipore). After blocking with 5% non‐fat milk for 1 hour, the membrane was incubated with specific primary antibodies (Supplementary Table 2) at 4°C overnight. The next day washed the membrane and then incubated with secondary antibodies (Zhuangzhi Biology, dilution rate of 1:5000) for 1 hour at room temperature. Proteins bands were detected by using ECL immunoblotting kit (Millipore) according to the manufacturer's protocol.

### 2.8 Luciferase reporter assay

Bioinformatics tools were used to analyse the SNAI3‐AS1 binding sites on UPF1. Plasmid GV208‐SNAI3‐AS1‐WT (Genechem) was constructed by inserting the sequence of SNAI3‐AS1 into the GV208 vector, and plasmid GV208‐SNAI3‐AS1‐Mut (Genechem) was established by inserting SNAI3‐AS1 sequence with the UPF1 binding site muted by site‐specific mutagenesis. Cells were cultured in 96‐well plates and proliferated to 60%‐80% confluence before transfection. Plasmid SNAI3‐AS1‐WT or Mut together with UPF1 plasmid or negative control were cotransfected using the Lipofectamine 2000 reagent (Invitrogen). After transfection for 48 hours, the Dual Luciferase Assay Kit was conducted to examine the luciferase activity according to the manufacturer's instructions. Renilla luciferase activity was used as control.

### 2.9 RNA immunoprecipitation (RIP)

RIP assays were performed using a Millipore EZ‐Magna RIP RNA‐Binding Protein Immunoprecipitation kit (Millipore) according to the manufacturer's protocol. Antibodies used for RIP included rabbit polyclonal IgG (Millipore) and antibodies to UPF1 (Abcam). RIP‐PCR was performed as qRT‐PCR using total RNA as input controls.

### 2.10 Immunofluorescence (IF)

Cells were cultured on glass coverslips for 24 hours to confluence, then fixed in 4% paraformaldehyde at room temperature for 15 minutes, and then permeabilized using 0.5% Triton X‐100 and blocked for 1 hour with 10% goat serum. Following incubated cells with primary antibody 4°C overnight and secondary antibodies with a appropriate dilution for 1 hour (Supplementary Table 2). After washing with PBS, DAPI was applied for nucleus staining. Images were collected using invert fluorescent microscope (Leica). Antibodies used in this study were presented in Supplement Table 2.

### 2.11 Statistical analysis

All data were analysed using SPSS 23.0 and Graphpad Prism 7.0. The difference between two groups was compared by Student's *t* test. Correlation between two groups was analysed using Pearson's correlation coefficient analysis. The Kaplan‐Meier test was used to assess prognosis. A value of *P* < 0.05 was considered significant.

## 3 RESULTS

### 3.1 SNAI3‐AS1 is up‐regulated in HCC and correlated with poor progression

**Figure 1:**
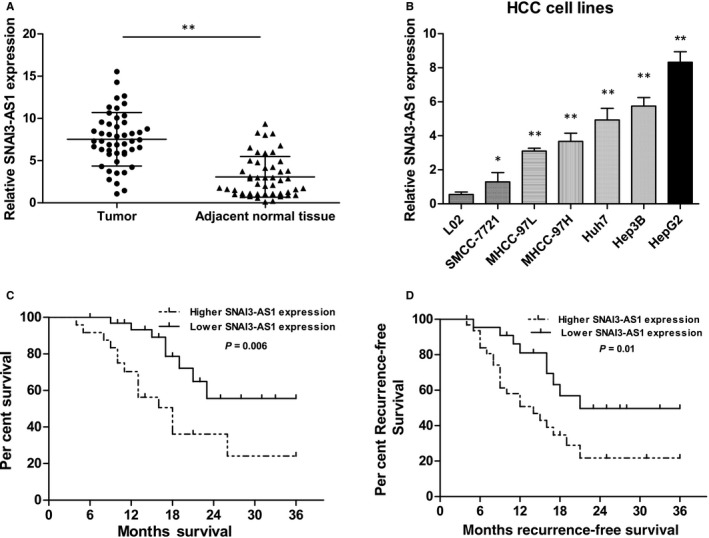
SNAI3‐AS1 was overexpressed in HCC tissues and cell lines. (A) qRT‐PCR analysis of SNAI3‐AS1 expression in 46 patients with HCC and matched adjacent normal tissues. **P < 0.01. (B) qRT‐PCR analysis of SNAI3‐AS1 expression in HCC cell lines and the immortalized human hepatic cell line LO2, *P < 0.05; **P < 0.01. (C,D) Kaplan‐Meier analysis of OS and RFS based on SNAI3‐AS1 expression levels in HCC patients. The median level of SNAI3‐AS1 is used as the cut‐off. Patients with HCC are divided into a high‐expression group and a low‐expression group

To investigate the roles of SNAI3‐AS1 in HCC, SNAI3‐AS1 expression was examined in HCC tissues and cell lines. QRT‐PCR revealed that expression of SNAI3‐AS1 was significantly elevated in 69.5% (32/46) of HCC tissues when compared with matched adjacent normal tissues (Figure 1A). To understand the significance of SNAI3‐AS1 overexpression in HCC, we investigated the potential associations between SNAI3‐AS1 expression and patients' clinicopathological features. Clinicopathological features of HCC patients were shown in Table 1. Noticeably, high SNAI3‐AS1 expression was significantly correlated with tumour size and advanced TNM stage. However, SNAI3‐AS1 expression was not associated with other parameters such as age, gender, AFP, HBV infection and Histologic grade in HCC. In addition, the result showed that SNAI3‐AS1 expression is higher in the HCC cell lines compared with the immortalized normal human hepatic cell LO2 (Figure 1B). To analyse the relationship between SNAI3‐AS1 expression and HCC patients overall survival (OS) or disease‐free survival (DFS), we performed Kaplan‐Meier and log‐rank analyses. Patients with higher SNAI3‐AS1 expression had shorter OS and DFS than patients with low levels of SNAI3‐AS1 (Figure 1C,D). These data showed that up‐regulation of SNAI3‐AS1 is associated with the development and progression of HCC.

**Table 1:** Relationship between SNAI3‐AS1 expression and clinical characteristics of HCC patients

Clinical factors	No. of cases	SNAI3‐AS1 expression		*P* value
		Low (n = 18)	High (n = 28)	
Age (y)				
＜59	19	10	9	0.116
≥59	27	8	19	
Gender				
Male	37	13	24	0.456
Female	9	5	4	
HBV infection				
Positive	34	12	22	0.580
Negative	12	6	6	
Tumour size				
<5 cm	24	15	9	0.001*
≥5 cm	22	3	19	
AFP (μg/L)				
<400	16	8	8	0.206
≥400	32	10	22	
Histologic grade				
Well & moderate	26	13	13	0.085
Low	20	5	15	
TNM stage				
I/II	31	16	15	0.013*
III/IV	15	2	13	

* Indicated statistical significance *P* < 0.05.

### 3.2 Knockdown of SNAI3‐AS1 represses the proliferation, invasion and migration of HCC cells in vitro

**Figure 2:**
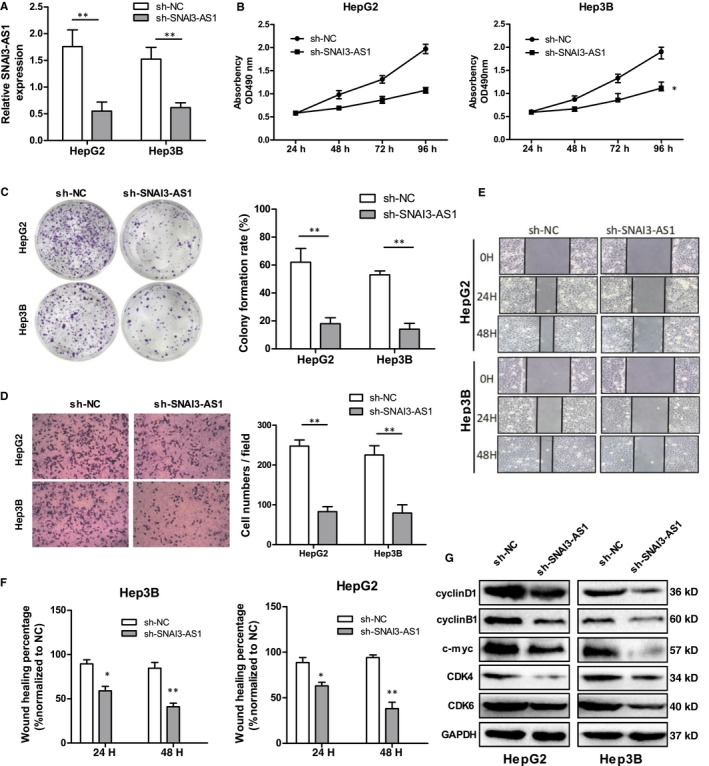
Down‐regulation of SNAI3‐AS1 inhibits HCC cell proliferation, invasion and migration in vitro. (A) qRT‐PCR analysis of SNAI3‐AS1 expression following transfected HCC cells with SNAI3‐AS1‐shRNAs, **P < 0.01. (B) MTT assays showing that silencing of SNAI3‐AS1 inhibited the proliferation of HCC cells. *P < 0.05. (C) SNAI3‐AS1 knockdown caused a decrease in the clonogenic survival of HCC cells. **P < 0.01. (D) Transwell assays showed SNAI3‐AS1 knockdown reduced the invasion of HCC cells. (scale bars = 50 mm). **P < 0.01. (E,F) Representative images of wound healing assays after SNAI3‐AS1 silencing. *P < 0.05; **P < 0.01. (G) Common cell proliferation‐related proteins expression levels detected by Western blot analysis following SNAI3‐AS1 silencing

In vitro experiments were performed to determine the effect of SNAI3‐AS1 on HCC cells proliferation and metastasis. Firstly, we reduced SNAI3‐AS1 expression with lentiviral shRNA‐SNAI3‐AS1 in HepG2 and Hep3B cells (Figure 2A). MTT and plate colony formation assays showed that knockdown of SNAI3‐AS1 impaired HepG2 and Hep3B cells proliferation (Figure 2B,C). To further examine the effects of SNAI3‐AS1 on HCC metastasis, transwell and wound healing assays were used to detected cell invasion and migration. Transwell assay with Matrigel indicated that down‐regulation of SNAI3‐AS1 inhibited the invasive activity of HepG2 and Hep3B cells (Figure 2D). The wound healing assay showed that suppression of SNAI3‐AS1 expression in HepG2 and Hep3B cells resulted in slower wound closure than that in negative control cells (Figure 2E,F). In addition, SNAI3‐AS1 knockdown inhibited the expression of CDK4, CDK6, c‐myc and two cell cycle associated protein cyclin B1 and cyclin D1 (Figure 2G). Taken together, SNAI3‐AS1 knockdown inhibited proliferation and metastasis of HCC cells in vitro.

### 3.3 SNAI3‐AS1 overexpression promotes the proliferation, invasion and migration of HCC cells in vitro

**Figure 3:**
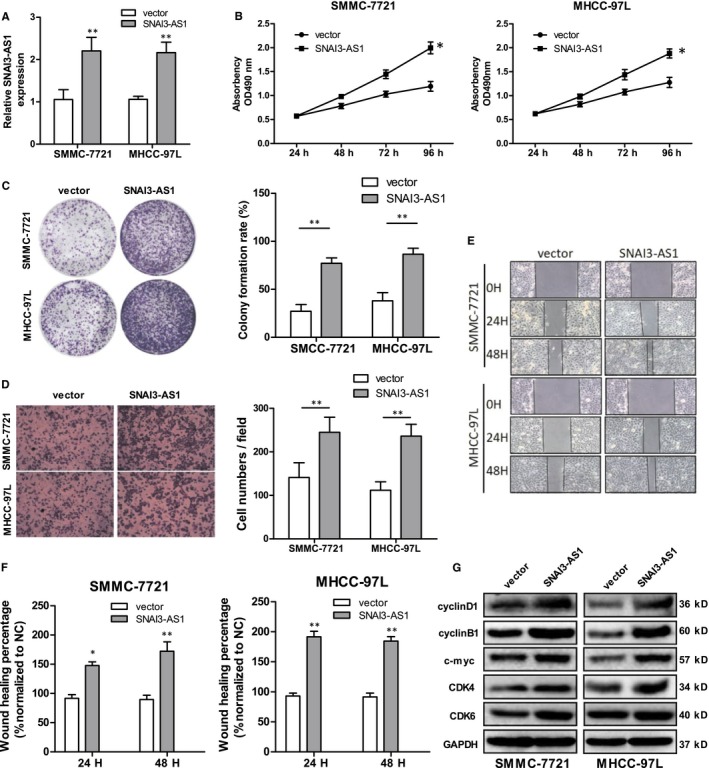
Overexpression of SNAI3‐AS1 promotes HCC cell proliferation, invasion and migration in vitro. (A) qRT–PCR analysis of SNAI3‐AS1 expression after SNAI3‐AS1 overexpression. **P < 0.01. (B) MTT assay showed SNAI3‐AS1 overexpression promotes HCC cells proliferation. *P < 0.05. (C) Overexpression SNAI3‐AS1 caused a decrease in the clonogenic survival of HCC cells. **P < 0.01. (D) Transwell assays showed overexpression SNAI3‐AS1 reduced the invasion of HCC cells. (scale bars = 50 mm). **P < 0.01. (E,F) Representative images of wound healing assays after SNAI3‐AS1 up‐regulation. *P < 0.05; **P < 0.01. (G) Common cell proliferation‐related proteins expression levels detected by Western blot analysis following SNAI3‐AS1 up‐regulation

We further examined the role of SNAI3‐AS1 by assessing the effect of its overexpression in SMMC‐7721 and MHCC‐97L cells, which have lower endogenous SNAI3‐AS1 levels. Firstly, a pCMV‐SNAI3‐AS1 expression plasmid was transfected to increase the expression of SNAI3‐AS1 in SMMC‐7721 and MHCC‐97L cells (Figure 3A). Growth curves produced by MTT assay showed that SNAI3‐AS1 up‐regulation significantly increased SMMC‐7721 and MHCC‐97L cells growth (Figure 3B). Consistently, the plate colony formation assays showed SNAI3‐AS1 overexpression caused an increase in the clonogenic survival of SMMC‐7721 and MHCC‐97L cells (Figure 3C). Meanwhile, transwell and wound healing assays were used to analyse differences in cell invasion and migration following SNAI3‐AS1 overexpression. Results showed SNAI3‐AS1 up‐regulation significantly promoted the invasion and migration of SMCC‐7721 and MHCC‐97L cells compared with empty vector (Figure 3D‐F). In addition, SNAI3‐AS1 up‐regulation increased the expression of CDK4, CDK6, c‐myc, cyclin B1 and cyclin D1, which related to cell growth (Figure 3G). Overall, SNAI3‐AS1 overexpression promoted the proliferation and metastasis of HCC cells in vitro.

### 3.4 SNAI3‐AS1 promoted HCC tumorigenesis by interacts with UPF1

**Figure 4:**
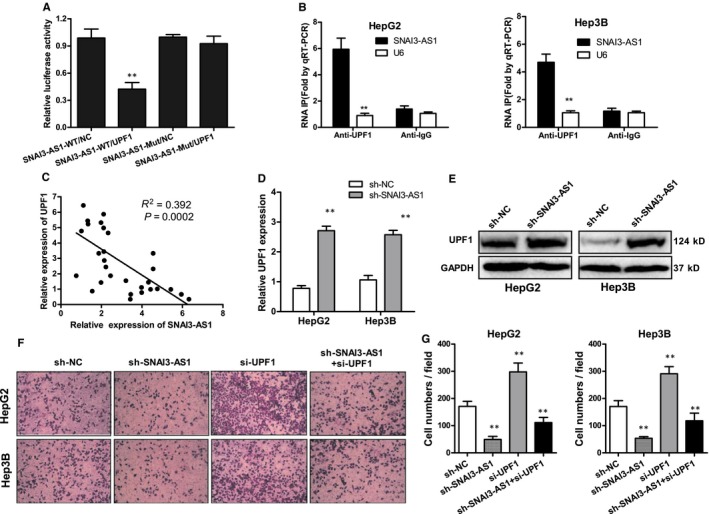
SNAI3‐AS1 promoted HCC tumorigenesis by binding UPF1. (A).Luciferase reporter assay was applied to verify the targeted binding effect between SNAI3‐AS1 3′UTR and UPF1. **P < 0.01. (B) HepG2 and Hep3B cells were harvested for RIP with an anti‐UPF1 antibody or control IgG. SNAI3‐AS1 levels were analysed by qRT‐PCR. **P < 0.01. (C) Pearson's correlation analysis of the relationship between UPF1 and SNAI3‐AS1 expression levels in HCC tissues. (D) qRT‐PCR analysis of UPF1 mRNA expression following SNAI3‐AS1 silencing **P < 0.01. (E) Western blot analysis of UPF1 protein expression following SNAI3‐AS1 silencing. (F,G) Transwell invasion assays performed using HepG2 and Hep3B cells after cotransfection with UPF1 siRNA and SNAI3‐AS1‐shRNA. **P < 0.01

Recently, several studies have found that many lncRNAs are involved in multiple regulation pathways through their interaction with RNA‐binding proteins (RBPs). To test this hypothesis, we sought to identify proteins that are associated with SNAI3‐AS1, and starBase and DIANA LncBase software were used for biological information prediction. The result showed that SNAI3‐AS1 contains a potential binding site for UPF1. UPF1 is known to interact with many RNA substrates and promote mRNA stability.^17^ Next, we constructed luciferase reporter vectors of SNAI3‐AS1, and luciferase reporter assay result showed cotransfection cells with pCMV‐SNAI3‐AS1‐WT and UPF1 vector significantly inhibited luciferase reporter activity, however, pCMV‐SNAI3‐AS1‐Mut in UPF1 putative targeting sites did not resulted in these effects (Figure 4A). To further validate the interaction between SNAI3‐AS1 and UPF1, we performed RNA immunoprecipitation (RIP) with an antibody against UPF1 using cell extracts from HepG2 and Hep3B HCC cell lines. We observed an enrichment of SNAI3‐AS1 with UPF1 antibody as compared to the non‐specific antibody (IgG control; Figure 4B). Meanwhile, Pearson's correlation analysis suggested that UPF1 expression was inversely correlated with SNAI3‐AS1 in HCC tissues (Figure 4C), and knockdown of SNAI3‐AS1 could increase UPF1RNA and protein levels in HepG2 and Hep3B cells (Figure 4D,E). We further studied the roles of SNAI3‐AS1 and UPF1 in HCC cell invasion using transwell invasion assay. The results showed that UPF1 suppressed HepG2 and Hep3B cells invasion, whereas SNAI3‐AS1 promoted invasion. However, the inhibitory effect of SNAI3‐AS1‐shRNA on HCC cell invasion could be partially restored by UPF1 inhibition (Figure 4F,G). These observations suggest that SNAI3‐AS1 knockdown could suppress HCC cell invasion by regulating UPF1 expression.

### 3.5 SNAI3‐AS1 induced EMT in HCC cells

**Figure 5:**
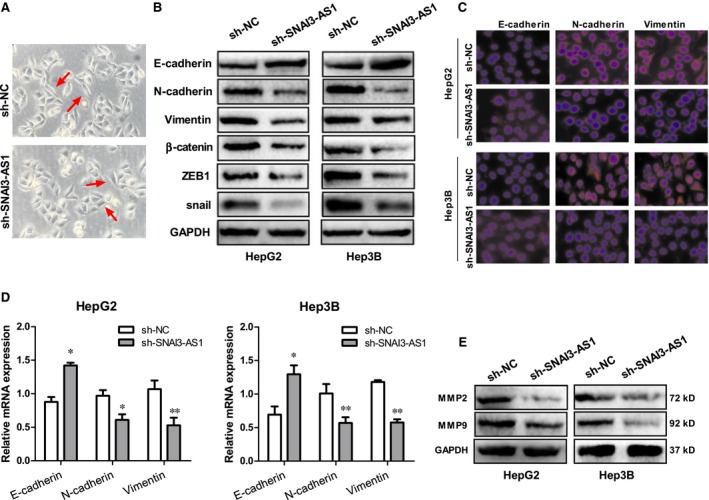
SNAI3‐AS1 induced EMT in HCC cells. (A) Knockdown of SNAI3‐AS1 induced the reversion of HepG2 cell morphological feature. (B) Western blot analyse the EMT relative protein markers in SNAI3‐AS1‐knockdown HepG2 and Hep3B cells. (C) IF analyse the EMT relative protein markers in SNAI3‐AS1‐knockdown HepG2 and Hep3B cells. (D) qRT‐PCR analyse the EMT markers mRNA in SNAI3‐AS1‐knockdown HepG2 and Hep3B cells. (E) Knockdown of SNAI3‐AS1 significantly reduced the expression of two matrix metalloproteinases MMP‐2 and MMP‐9

Since increasing evidence identified that tumour migration and invasion is tightly involved in EMT, so we speculated that SNAI3‐AS1 induces EMT in HCC cells. Firstly, we examined the effect of SNAI3‐AS1 on cell phenotype. Knockdown of SNAI3‐AS1 induced the reversion of mesenchymal‐like morphological feature into an epithelial phenotype in HepG2 cells (Figure 5A). Western blot and qRT‐PCR analysis indicated that epithelial marker E‐cadherin showed increased expression, whereas mesenchymal markers (N‐cadherin and vimentin) and some EMT relative protein markers showed decreased expression in SNAI3‐AS1‐knockdown HepG2 and Hep3B cells (Figure 5B,D). To further explore the effect of targeting SNAI3‐AS1 on EMT, IF was employed to analyse the expression of EMT markers in HepG2 and Hep3B cells. As shown in Figure 5C, SNAI3‐AS1‐shRNA increased expression of E‐cadherin but decreased expression of N‐cadherin and vimentin compared with negative control cells. In addition, knockdown of SNAI3‐AS1 significantly reduced the expression of two matrix metalloproteinases MMP‐2 and MMP‐9 that are closely correlated with metastasis (Figure 5E). Overall, these data indicated that SNAI3‐AS1 induced EMT in HCC cells.

### 3.6 SNAI3‐AS1 activates the TGF‐β/Smad pathway by binding UPF1

**Figure 6:**
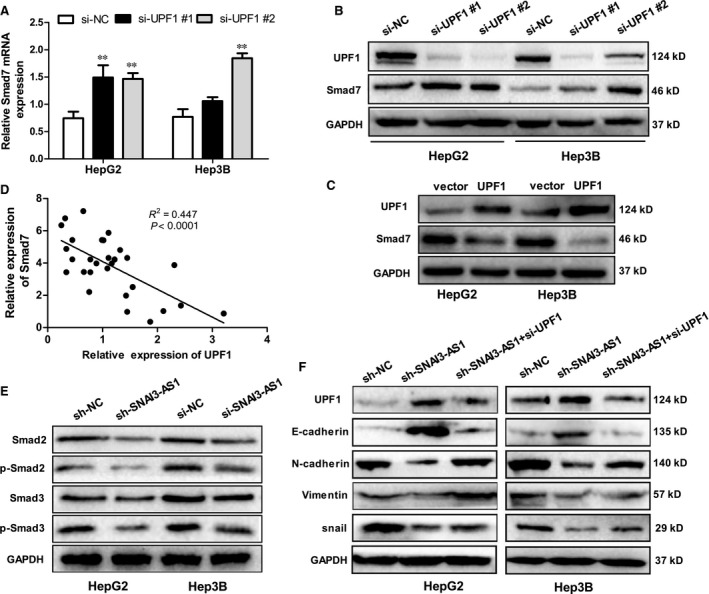
SNHG6 induced EMT by binding UPF1 and activating the TGF‐β/Smad pathway. (A) qRT‐PCR analysis of Smad7 mRNA expression following UPF1 silencing **P < 0.01. (B) Western blot analysis of Smad7 protein expression following UPF1 silencing. (C) Western blot analysis of Smad7 protein expression following UPF1 up‐regulation. (D) Pearson's correlation analysis of the relationship between UPF1 and Smad7 expression levels in HCC tissues. (E) The expression of total and phosphorylation of key molecules of the TGF‐β pathways (Smad2, Smad3) were detected using Western blot. (F) The EMT markers were detected in HepG2 and Hep3B cells after cotransfection with UPF1 siRNA and control (scramble) or SNAI3‐AS1 shRNA

**Figure 7:**
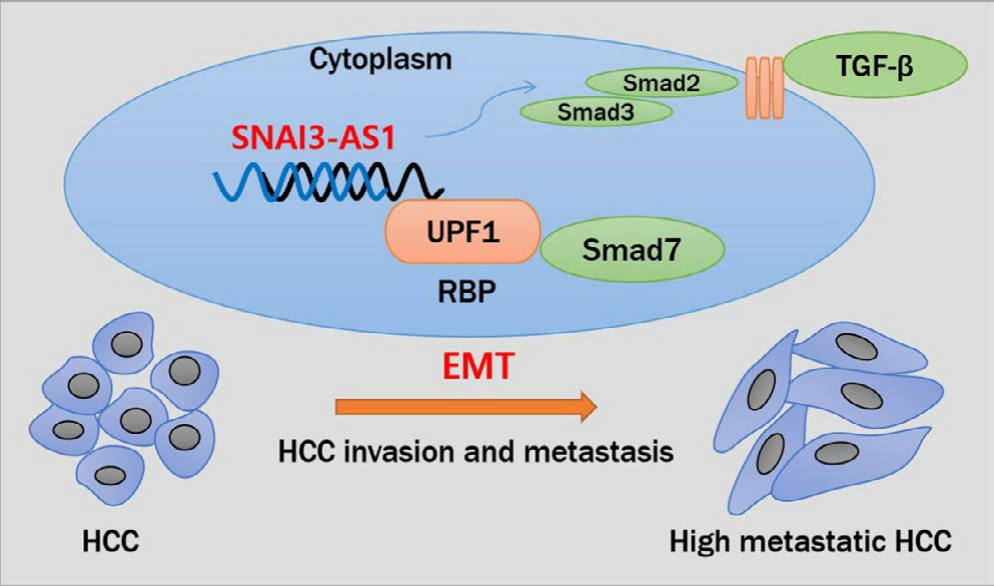
A schematic model depicting the functions of SNAI3‐AS1 in HCC. SNAI3‐AS1 induced EMT by binding UPF1, regulating Smad7 expression, and inducing activation of the TGF‐β/Smad pathway

Study had shown that UPF1 could promote TGF‐β signalling and inhibits neural differentiation by targeting Smad7 mRNA,^16^ thus, we hypothesize that SNAI3‐AS1 promotes HCC cell invasion by regulating the ability of UPF1 to mediate the TGF‐β/Smad pathway. To further study the relationship between UPF1 and Smad7, two specific siRNAs ( siRNA #1,#2) against UPF1 gene transcript were introduced into HepG2 and Hep3B cells, and produced the greatest reduction in endogenous UPF1 expression (Figure 6A). Western blotting confirmed that Smad7 expression level was up‐regulated when UPF1 was silenced in HCC cells (Figure 6B). While the Smad7 expression level was down‐regulated when UPF1 was overexpressed in HCC cells (Figure 6C). Pearson's correlation analysis showed that Smad7 mRNA expression was significantly negatively correlated with UPF1 in HCC tissues (Figure 6D). Previous studies had demonstrated the key role of Smad7 in the TGF‐β pathway. As shown in our study, UPF1 could affect the expression of Smad7 in HCC. Therefore, we speculated that SNAI3‐AS1 could affect the TGF‐β pathway by regulating UPF1. As shown in Figure 6E, SNAI3‐AS1 knockdown significantly decreased phosphorylation of Smad2/3, whereas total Smad2/3 expression level was similar between groups. In addition, the rescue experiments were conducted to prove that SNAI3‐AS1 promotes tumour EMT by regulating UPF1. As shown before, knockdown of SNAI3‐AS1 increased epithelial marker E‐cadherin and decreased mesenchymal markers N‐cadherin and vimentin, however, these effects could be partially restored by UPF1 inhibition (Figure 6F). All these results demonstrated that SNAI3‐AS1 promotes HCC tumorigenesis by binding UPF1, regulating Smad7 expression, and inducing activation of the TGF‐β/Smad pathway (Figure 7).

## 4 DISCUSSION

Genomic studies revealed that less than 2% of the human genome has protein‐coding functions.^18^ High‐resolution microarrays and massively parallel sequencing technologies played a key role in identifying and elucidating non‐coding RNAs (ncRNAs). Long non‐coding RNAs (lncRNAs), long than 200 nucleotides, represented a vital proportion in ncRNAs. The flexibility of the location and distribution of lncRNAs in the genome allowed them to interact directly or indirectly with proteins, RNA or more unknown molecules, thereby broadly participating in gene regulation, cancer phenotype, cell differentiation and even chromatin remodelling.^19^, ^20^, ^21^ LncRNA had dramatically altered our understanding of the biology of complex diseases, including cancers. Increasing evidence correlated changes in expression levels of lncRNAs to cancers, and lncRNA expression profiling was an approach for identification of molecular biomarkers for tumour occurrence, classification and prognosis.^17^, ^22^ In terms of gene therapy, various therapeutic approaches for the lncRNAs have been developed, for example, the siRNAs against the specific lncRNA can be used to modulate lncRNA function. Furthermore, antisense oligonucleotides (ASOs) can be adapted to directly target lncRNAs when the overall secondary structure or the nucleotide sequence is detrimental to the optimal design of the siRNA.^23^, ^24^ Therefore, lncRNAs can potentially be used as diagnostic markers or therapeutic targets.

Recently, increasing evidence revealed functional roles of lncRNAs in metastasis and progression of HCC.^25^, ^26^ For instance, CDKN2B‐AS1, an oncogenic lncRNA of HCC, promoted NAP1L1‐mediated PI3K/AKT/mTOR signalling by acting as a molecular sponge of let‐7c‐5p, which indicated that CDKN2B‐AS1 may be a potential prognostic biomarker and a candidate target for HCC therapy.^27^ In the present study, we focused on lncRNA SNAI3‐AS1 (NR_015378), which located at Xp11.23, is a novel lncRNA which identified from a lncRNA microarray analysis. We first reported that the high expression of SNAI3‐AS1 in HCC, and combined with the clinical and pathological characteristics of HCC patients, we determined that high SNAI3‐AS1 expression was significantly associated with tumour sizes and TNM stage. More importantly, high levels of SNAI3‐AS1 were associated with poor prognosis in patients with HCC. We further observed that overexpression of SNAI3‐AS1 promoted HCC cell proliferation and migration in vitro, while knockdown of SNAI3‐AS1 had the opposite effect. Therefore, our finding indicated that SNAI3‐AS1 may act as a tumour oncogene in HCC.

Research confirmed that lncRNA regulated gene transcription by interfacing with correspondent RNA‐binding proteins (RBPs) in a specific manner.^27^, ^28^ UPF1 is a key factor in nonsense‐mediated mRNA degradation (NMD) and is a highly conserved pathway for the selective degradation of aberrant RNA transcripts. At the same time, UPF1 can also function as RBP.^29^, ^30^ UPF1 not only acts on RNA degradation but also plays a key role in tumour progression.^31^, ^32^ Studies showed that UPF1 can induce the degradation of NMD substrate of Smad7 and stimulate TGF signalling, which may be one of the mechanisms regulating HCC metastasis.^33^ In this study, by bioinformatics analysis and follow‐up experimental verification, we identified a specific interaction between SNAI3‐AS1 and UPF1. Knockdown of SNAI3‐AS1 suppressed HCC cell invasion by regulating UPF1 expression. Our findings provided new insight to the mechanism of lncRNA in the development of HCC.

Tumour metastasis is an important factor in the malignant progression of tumorigenesis and tumour death. EMT is a key mechanism of tumour metastasis, regulated by different signalling pathways and various growth factors.^34^ TGFβ was the key EMT inducer and regulated various cellular responses.^35^, ^36^ In this study, SNAI3‐AS1 regulated EMT by targeting the TGFβ signalling pathway in two non‐mutually exclusive ways. Firstly, knockdown of SNAI3‐AS1 decreased the expression of Smad2/3 proteins and then inhibited the TGFβ signalling. Secondly, SNAI3‐AS1 affected the expression of Smad7 through UPF1, which was a key molecule of the TGFβ signalling. In addition, opposite results were obtained in response to the UPF1 knockdown. In part, SNAI3‐AS1 indirectly regulated Smad7 through UPF1 and influenced the TGFβ signal pathway.

## 5 CONCLUSION

In conclusion, the current study demonstrated that highly expressed SNAI3‐AS1 was a novel oncogene which promoted the tumorigenesis and progression of HCC through regulating UPF1. Down‐regulation of SNAI3‐AS1 inhibited proliferation and metastasis of HCC cells. Furthermore, functional analyses indicated that SNAI3‐AS1 promotes HCC metastasis by inducing EMT. Finally, we demonstrated that SNAI3‐AS1 indirectly regulates Smad7 through UPF1 and influenced the TGF‐β pathway. However, further verification by a large number of clinical samples is still required in the future. Our research maybe a little part of the complicated mechanism on tumorigenesis of HCC, but it is worthwhile to reveal that SNAI3‐AS1 may be a novel prognostic factor and potential therapeutic target for HCC.

## CONFLICT OF INTEREST

The authors confirm that there are no conflicts of interest.

## AUTHORS' CONTRIBUTIONS

HSX and LGF conceived and designed the project. LYR and GD carried out most of the experiments. ZY, RMD and CYF helped to collect tissue samples. LYP and WX collected the clinical and pathological data. WX performed the statistical analysis. LYR wrote the manuscript, all authors read and approved the final manuscript.

## Data Availability

The data in this study are available.
