# Correction to Long non‐coding RNA SNAI3‐AS1 promotes the proliferation and metastasis of hepatocellular carcinoma by regulating the UPF1/Smad7 signalling pathway

**DOI:** 10.1111/jcmm.17769

**Published:** 2023-07-21

In Li Yarui et al.,^1^ incorrect images were used for sh‐NC and sh‐SNAI3‐AS1 + si‐UPF1 of HepG2 in Figure 4F due to technical error during image preparation. The correct Figure 4 is shown below. The authors confirmed that all results and conclusions of this article remain unchanged.

**FIGURE 4:**
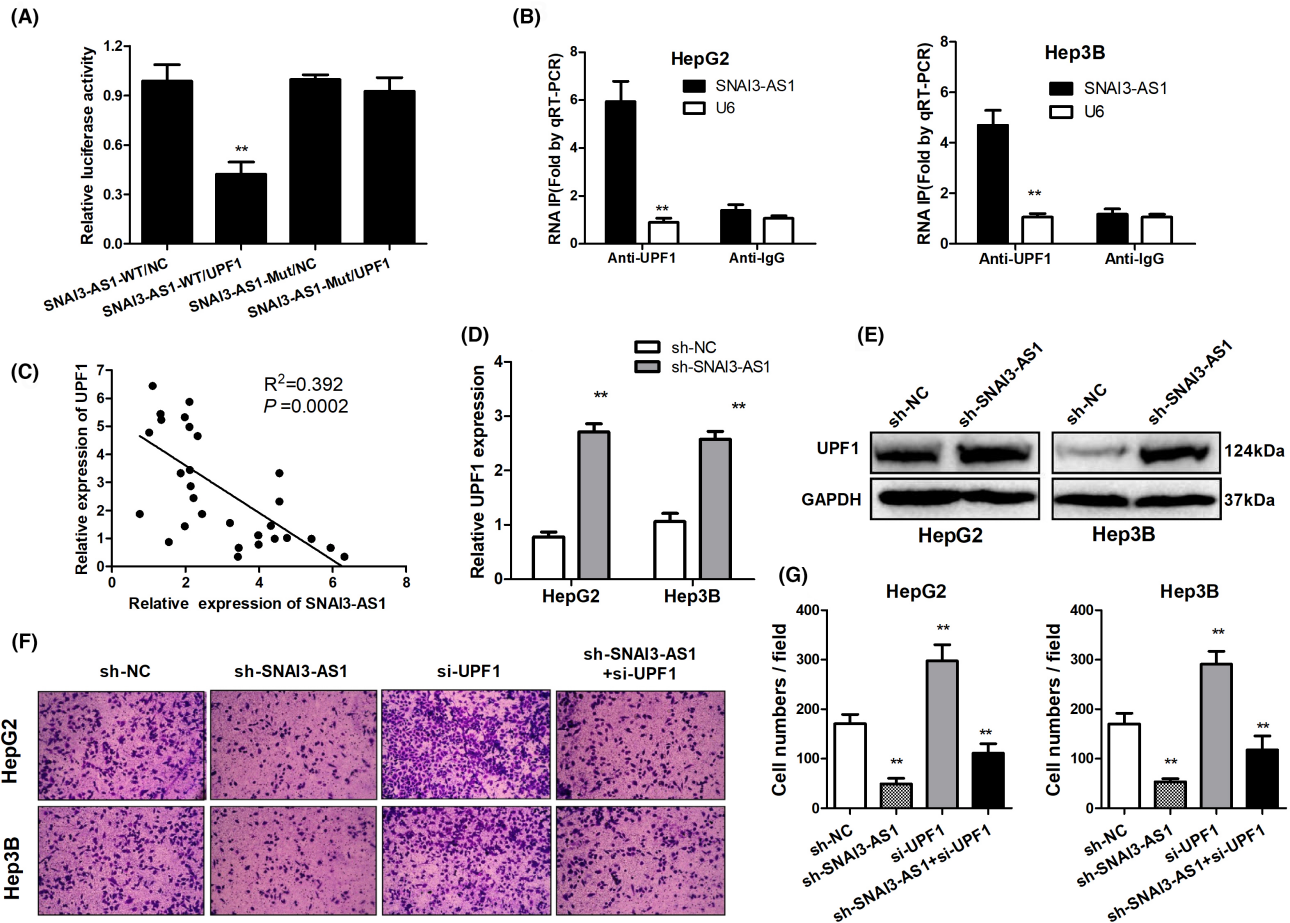
SNAI3‐AS1 promoted HCC tumorigenesis by binding UPF1. (A) Luciferase reporter assay was applied to verify the targeted binding effect between SNAI3‐AS1 3′UTR and UPF1. **p < 0.01. (B) HepG2 and Hep3B cells were harvested for RIP with an anti‐UPF1 antibody or control IgG. SNAI3‐AS1 levels were analysed by qRT‐PCR. **p < 0.01 (C) Pearson's correlation analysis of the relationship between UPF1 and SNAI3‐AS1 expression levels in HCC tissues (D). qRT‐PCR analysis of UPF1 mRNA expression following SNAI3‐AS1 silencing **p < 0.01. (E) Western blot analysis of UPF1 protein expression following SNAI3‐AS1 silencing. (F, G). Transwell invasion assays performed using HepG2 and Hep3B cells after cotransfection with UPF1 siRNA and SNAI3‐AS1‐shRNA. **p < 0.01.
