# Supplementary material for: Long non‐coding RNA SNAI3‐AS1 promotes the proliferation and metastasis of hepatocellular carcinoma by regulating the UPF1/Smad7 signalling pathway
Source: J Cell Mol Med. 2019 Jul 2;23(9):6271–82. doi: 10.1111/jcmm.14513 (PMC6714236; doi:10.1111/jcmm.14513)
Supplement: Supplementary file 1 [file JCMM-23-6271-s001.docx]

**Supplementary Tables**

**Supplementary Table 1. Primer sequences and target sequences used in this study**

| **Gene** | **Sequence or Target Sequence** |
| --- | --- |
| SNAI3-AS1-F | 5'-GCGTTATGTCGTTTGGTTGATG-3' |
| SNAI3-AS1-R | 5'-TGGCAGGAATGAGGTGAGC-3' |
| β-actin-F | 5'-ATCGTGCGTGACATTAAGGAGAAG-3' |
| β-actin-R | 5'AGGAAGGAAGGCTGGAAGAGTG-3' |
| UPF1-F | 5'-ACCACGAAGTTGCTGAAGG-3' |
| UPF1-R | 5'-ACACAGGACAGGATGATGAAG-3' |
| E-cadherin-F | 5'-ATTCTGATTCTGCTGCTCTTG-3' |
| E-cadherin-R | 5'-AGTCCTGGTCCTCTTCTCC-3' |
| N-cadherin-F | 5'-CATCATCCTGCTTATCCTTGTG-3' |
| N-cadherin-R | 5'-CATAGTCCTGGTCTTCTTCTCC-3' |
| Vimentin-F | 5'-GCTGGAAGGCGAGGCGAGGAGAG-3' |
| Vimentin-R | 5'-CAACCGTCTTAATCAGAAGTGTC-3' |
| Smad7-F | 5'-TTCAAGTAATCCAGGATAGGCT-3' |
| Smad7-R | 5'-CGGTAAGAAGATGGAACCATAA-3' |
| UPF1-siRNA #1 | 5'-CCCUGAUAAUUAUGGCGAUTT-3' |
| UPF1-siRNA #1 | 3'-AUCGCCAUAAUUAUCAGGGTT-5' |
| UPF1-siRNA #2 | 5'-CCUUCCCAUCCAACAUCUUTT-3' |
| UPF1-siRNA #2 | 3'-AAGAUGUUGGAUGGGAAGGTT-5' |
| siRNA-NC-F | 5'-UUCUCCGAACGUGUCACGUTT-3' |
| siRNA-NC-R | 3'-ACGUGACACGUUCGGAGAATT-5' |
| SNAI3-AS1-shRNA | 5'-GGUUAUGGGAAGUUUAAUATT -3' |
| SNAI3-AS1-shRNA | 3'-UAUUAAACUUCCCAUAACCTT -5' |

**Supplementary Table 2. Antibodies used in this study**

| **Antibody (Item No.)** | **Application** | | **Specificity** | | **Company** |
| --- | --- | --- | --- | --- | --- |
|  | **WB** | **IF** | |  |  |
| β-actin(13E5) | 1:1000 |  | | Rabbit monoclonal | Cell Signaling Technology |
| CDK4(sc-166373) | 1:500 |  | | Mouse monoclonal | Santa Cruz Biotechnology |
| CDK6(sc-7961) | 1:500 |  | | Mouse monoclonal | Santa Cruz Biotechnology |
| UPF1(ab109363) | 1:1000 |  | | Rabbit monoclonal | Abcam |
| Smad7(ab216428) | 1:500 |  | | Rabbit monoclonal | Abcam |
| CyclinB1(ab72) | 1:1000 |  | | Rabbit monoclonal | Abcam |
| CyclinD1(ab134175 ) | 1:1000 |  | | Rabbit monoclonal | Abcam |
| c-Myc(D3N8F) | 1:1000 |  | | Rabbit monoclonal | Cell Signaling Technology |
| MMP-2(D8N9Y) | 1:500 |  | | Rabbit monoclonal | Cell Signaling Technology |
| MMP-9(D6O3H) | 1:1000 |  | | Rabbit monoclonal | Cell Signaling Technology |
| E-cadherin(24E10) | 1:1000 | 1:200 | | Rabbit monoclonal | Cell Signaling Technology |
| N-cadherin(D4R1H) | 1:500 | 1:200 | | Rabbit monoclonal | Cell Signaling Technology |
| Vimentin(D21H3) | 1:1000 | 1:100 | | Rabbit monoclonal | Cell Signaling Technology |
| Snail (C15D3) | 1:1000 |  | | Rabbit monoclonal | Cell Signaling Technology |
| β-catenin(D10A8) | 1:1000 |  | | Rabbit monoclonal | Cell Signaling Technology |
| ZEB1(D80D3) | 1:1000 |  | | Rabbit monoclonal | Cell Signaling Technology |
| Smad2(ab40855) | 1:1000 |  | | Rabbit monoclonal | Abcam |
| p-Smad2(ab53100) | 1:500 |  | | Rabbit monoclonal | Abcam |
| Smad3(ab40854) | 1:1000 |  | | Rabbit monoclonal | Abcam |
| p-Smad3(ab52903) | 1:500 |  | | Rabbit monoclonal | Abcam |
